# Dermatan Sulfate-Free Mice Display Embryological Defects and Are Neonatal Lethal Despite Normal Lymphoid and Non-Lymphoid Organogenesis

**DOI:** 10.1371/journal.pone.0140279

**Published:** 2015-10-21

**Authors:** Xanthi N. Stachtea, Emil Tykesson, Toin H. van Kuppevelt, Ricardo Feinstein, Anders Malmström, Rogier M. Reijmers, Marco Maccarana

**Affiliations:** 1 Department of Experimental Medical Science, Lund University, Lund, Sweden; 2 Department of Biochemistry, Radboud Institute for Molecular Life Sciences, Radboud University Medical Center, Nijmegen, the Netherlands; 3 Department of Pathology, The National Veterinary Institute (SVA), SE 75189, Uppsala, Sweden; 4 Department of Molecular Cell Biology and Immunology, VU University Medical Center, Amsterdam, the Netherlands; Columbia University, UNITED STATES

## Abstract

The epimerization of glucuronic acid into iduronic acid adds structural variability to chondroitin/dermatan sulfate polysaccharides. Iduronic acid-containing domains play essential roles in processes such as coagulation, chemokine and morphogen modulation, collagen maturation, and neurite sprouting. Therefore, we generated and characterized, for the first time, mice deficient in dermatan sulfate epimerase 1 and 2, two enzymes uniquely involved in dermatan sulfate biosynthesis. The resulting mice, termed DKO mice, were completely devoid of iduronic acid, and the resulting chondroitin sulfate chains were structurally different from the wild type chains, from which a different protein binding specificity can be expected. As a consequence, a vast majority of the DKO mice died perinatally, with greatly variable phenotypes at birth or late embryological stages such as umbilical hernia, exencephaly and a kinked tail. However, a minority of embryos were histologically unaffected, with apparently normal lung and bone/cartilage features. Interestingly, the binding of the chemokine CXCL13, an important modulator of lymphoid organogenesis, to mouse DKO embryonic fibroblasts was impaired. Nevertheless, the development of the secondary lymphoid organs, including the lymph nodes and spleen, was normal. Altogether, our results indicate an important role of dermatan sulfate in embryological development and perinatal survival.

## Introduction

Proteoglycans (PGs) are important constituents of the cell membrane and the extracellular matrix (ECM) and are involved in the orchestration of a vast variety of important biological processes [1, 2]. PGs are composed of a protein core with covalently attached glycosaminoglycan (GAG) polysaccharide chains, such as chondroitin/dermatan sulfate (CS/DS). Chondroitin consists of alternating N-acetylgalactosamine (GalNAc) and glucuronic acid (GlcA) residues. GlcA can be converted into iduronic acid (IdoA) by DS epimerase-1 (DS-epi1) and DS epimerase-2 (DS-epi2) (encoded by *Dse* and *Dsel*, respectively) [3]. Following epimerization and sulfation at different positions, copolymeric CS/DS is formed, in which IdoA can be found in different patterns: in long blocks of consecutive IdoA residues (n ≥6), dispersed in the chain as single IdoA, or as alternating IdoA/GlcA structures. *In vivo*, DS-epi1 is mainly responsible for the generation of long blocks of IdoA, while DS-epi2 mainly produces single or alternating structures [4]. In hybrid CS/DS chains, the extent of IdoA is variable, ranging from one IdoA residue per chain to almost 100% IdoA, depending on the proteoglycan, the tissue where it is expressed and the physiological conditions. DS-epi1 is the major epimerase in most tissues, while DS-epi2 is dominant in brain tissue. Interestingly, DS-epi1 deficient mice revealed a fragile skin phenotype, a smaller body size and several malformations [5, 6]. In contrast, the loss of DS-epi2 produced completely normal offspring [7]. These findings suggest that although tissue-specific expression of DS-epi1 and DS-epi2 is observed, redundancy cannot be ruled out in these single deficient mice.

The biological functions of CS/DS are highly specialized and depend on the different IdoA-containing structural micro-domains that can specifically interact with morphogens, growth factors, cytokines, chemokines, adhesion molecules and lipoproteins, thereby regulating cell proliferation, cell signaling, blood coagulation and neurite outgrowth [8], processes that are essential during organogenesis. Interestingly, studies of a mouse model with inactivation of the single heparan sulfate (HS) epimerase *Glce* gene revealed kidney agenesis, lung defects, skeletal malformations and impaired lymphoid organ development [9, 10]. In addition, we previously demonstrated that lymphoid and non-lymphoid organs, including the spleen, lung and kidney, exhibit very high DS epimerase activity. Altogether, these findings prompted us to study the role of DS in embryonic development and (lymphoid) organogenesis. For this reason, we generated double-knock out mice, hereafter termed DKO, lacking both DS-epi1 and DS-epi2 that can serve as a DS-free *in vivo* model.

Here, we show that the loss of DS results in embryological developmental defects and neonatal lethality, despite the absence of major aberrations in lymphoid and non-lymphoid organogenesis.

## Materials and Methods

### Ethics statement

Permission for all animal experiments was granted by the regional ethical committee and all experiments performed were in compliance with national guidelines (Lund, Sweden, permit numbers: M439-12, M281-12).

### Mice

Mice deficient in *Dse* and *Dsel* in the mixed C57BL/6-129/SvJ genetic background were described previously [5, 7]. *Dse* ^+/-^ *Dsel* ^*-/-*^ mice were mated in order to obtain *Dse* ^+/+^ *Dsel* ^*-/-*^, *Dse* ^+/-^ *Dsel* ^*-/-*^ and *Dse* ^-/-^ *Dsel* ^*-/-*^ (DKO) mice. Pregnant *Dse* ^+/-^ *Dsel* ^*-/-*^ mice were sacrificed and E13.5, E16.5, E18.5 and E19.5 embryos were dissected and evaluated macroscopically and their body weight was measured. Organs were dissected from E18.5 embryos for immunohistochemistry. Mice were genotyped using PCR on extracted DNA from tail-tips. The following primers were used for *Dse*: forward primer for both alleles, 5′-AGCACATTGCAGCTCGGCTTAC-3′; reverse primer for the wild-type allele, 5′-GCTGCCATCCTCTCCATGTAGTC-3′; reverse primer for the neomycin cassette-mutated allele, 5′-TGGATGTGGAATGTGTGCGAGG-3′ and for *Dsel*: forward primer for both alleles: 5′-ACGTGGTCAAATGGCTTCAT-3′; reverse primer for the wild-type allele: 5′- GCTGTGAAATCCAGGTGACAT-3′; reverse primer hybridizing to the neomycin cassette: 5′-ATTAAGGGCCAGCTCATTCC-3′.

### Antibodies

For immunofluorescence and/or flow cytometry the following antibodies were used: GK1.5 (anti-CD4), MP33 (anti-CD45), MECA-367 (anti-mucosal addressin cell adhesion molecule-1 [MAdCAM-1]), and ERMP-12 (anti-CD31), which were purified from hybridoma cell culture supernatants by affinity chromatography with protein G-Sepharose (Pharmacia Biotech, Uppsala, Sweden) and labeled with Alexa Fluor-488, -555, or -647 (all from Invitrogen Life Technologies, Breda, the Netherlands). 145–211 (anti-CD3e, eBioscience, San Diego, CA, USA) Alexa Fluor-555 or Pe-labeled, and TUJ1 (anti-neuronal class III β-tubulin, BioLegend, Dedham, MA, USA) Alexa Fluor-488 labeled. Phage-display-derived single-chain antibodies vesicular stomatitis virus-tagged GD3A12 [11] and HS4E4 [12] were used and detected by a Cy3-conjugated secondary antibody anti-vesicular stomatitis virus, P5D4 (Sigma-Aldrich, St. Louis, MO, USA). To assure specificity of the antibodies, conjugate-alone controls as well as control serum (rat or rabbit) as replacement of the primary incubation were used. For western blots an anti-mouse decorin rabbit polyclonal was used at 1:1,000 dilution (immunogen: rat decorin; kindly provided by Åke Oldberg).

### Preparation and analysis of *in vivo* labeled CS/DS

Two-day-old pups from two litters derived from *Dse* ^+/-^ *Dsel* ^***-/-***^ parents were intraperitoneally injected with 0.5 mCi ^35^S-sodium sulfate (1,500 Ci/mmol from PerkinElmer) in 40 μl PBS and kept warm for 90 min before euthanasia. Following genotyping, two DKO, four *Dse* ^+/-^ *Dsel* ^***-/-***^, and two *Dse* ^+/+^ *Dsel* ^***-/-***^ mice were pooled. The CS/DS preparations came from the skin and from the whole remaining parts of the body.

The skin was removed and Potter homogenized in buffer containing 4 M guanidine, 50 mM acetate (pH 5.5), 10 mM EDTA, 10 mM NEM, 1 mM PMSF, and 0.1% Triton X-100. The extract was dialyzed versus 6 M urea, 50 mM acetate, pH 5.5, 0.2 M NaCl, and 1 mM EDTA. After the addition of 0.1% Triton, the extract was bound to DE-52 anion exchange resin, which was washed with the urea-containing buffer and eluted with the guanidine-containing buffer. PGs were size separated on Superose 6 columns run in guanidine-containing buffer into large PGs including versican and small PGs including decorin and biglycan [5]. The recovered PGs were protease-digested, the HS chains were degraded by deamination at pH 1.5, and the CS/DS labeled chains were recovered from Superose 6 columns run in 0.2 M ammonium bicarbonate.

The remaining whole body was Potter homogenized in proteinase K buffer (100 mM Tris, pH 8.5, 200 mM NaCl, 5 mM EDTA, and 0.2% SDS containing 100 μg/ml of proteinase K) and incubated overnight at 55°C. Labeled CS/DS were purified on DE-52 gels and recovered from degraded HS as outlined above for the skin CS/DS preparation.

The purity of labeled CS/DS was ascertained by quantitative degradation to disaccharides by chondroitinase ABC (Sigma C3667). Quantification and spatial arrangement of IdoA along the chain was analyzed by cleaving 40,000 dpm of CS/DS with 2 mIU of chondroitinase B (Seikagaku) in 20 mM Hepes, pH 7.2, 50 mM NaCl, 4 mM CaCl_2_, and 0.1 mg/ml BSA for 90 min. The split products were separated on a Superdex Peptide column (HealthCare) run in 0.2 M ammonium bicarbonate [5].

### Preparation of unlabeled skin PGs

Lyophilized E19.5 embryo skin samples were disrupted with TissueLyser II (Qiagen). PGs were extracted with the guanidine-containing buffer as above, which was later exchanged for the urea-containing buffer, and purified by DE-52 anion exchange resin as above, eluting them in 2M NH4HCO3, which was subsequently removed by lyophilization.

### Disaccharide fingerprint

The organs from three E19.5 embryos of each genotype: *Dse* ^+/+^ *Dsel* ^*+/+*^, *Dse* ^+/+^ *Dsel* ^*-/-*^, *Dse* ^+/-^ *Dsel* ^*-/-*^, and from two *Dse* ^-/-^ *Dsel* ^*-/-*^ embryos were pooled and dried by lyophilization. The proteins were degraded in an o.n. incubation at 55°C with 200 μg/ml pronase in pronase buffer (50 mm Tris/HCl, pH 8, 1 mM CaCl_2_, and 1% Triton X-100). After heat-inactivation of the protease, MgCl_2_ was added to 2 mM and benzonase was added to a final 20 Sigma units/ml and incubated for 2 h at 37°C. DNAase was heat-inactivated, NaCl was added to a final 0.1 M, and GAGs were purified by batch incubation with a DE52 anion exchange gel, followed by the transfer of the gel to disposable spin centrifuge tubes (Costar 8163). The tubes were washed with buffer 1 (50 mm Tris/HCl, pH 8, 0.1 m NaCl, and 0.1% Triton X-100), buffer 2 (50 mm NaAc, pH 4, 0.1 m NaCl, and 0.1% Triton X-100), water, and 0.1 M NH_4_HCO_3_. Elution was achieved by 2 M NH_4_HCO_3_. After lyophilization, GAGs were estimated by carbazole [13]. Then, 500 ng GAGs were subjected to chondroitinase ABC degradation (overnight at 37°C in 20 μl 50 mM NH_4_OAc and 0.1 mg/ml BSA, containing 10 mIU ABC). The samples were boiled and the supernatant was dried. Fluorophore-labelling of the resulting disaccharides was performed by adding 10 μl of 20 mM re-purified 2-aminoacridone (AMAC, Sigma), followed by a 20 min incubation at room temperature before the addition of 10 μl of 1 M NaBH_3_CN and incubation at 45°C for 16 h (modified from [14]). The re-purification of AMAC was achieved by reversed-phase chromatography on a Zorbax RX-C8 semi-preparative column (9.4x250 mm, 5 μm, Agilent), using a 13 min isocratic separation with 65% NH_4_OAc and 35% MeCN as eluents at 3 ml/min. To purify milligrams of AMAC, several 100 μl sandwich injections of the sample (0.1 M in DMSO) were made in 5% air, 10% DMSO, 70% sample, and 10% DMSO. Pre-column AMAC-labeled disaccharides were analyzed with HPLC-fluorescence as described previously, with slight modifications [14]. Briefly, 20 μl samples were diluted to 100 μl in running buffer (98% A: NH_4_OAc, 60 mM, pH 5.6, and 2% B: MeCN) and injected (2 μl) onto an XBridge BEH Shield RP18 (2.1x100 mm, 2.5 μm). Disaccharides were separated using a 39 min gradient run at 0.35 ml/min (0–1 min: 98% A, 1–3 min: 98–96% A, 3–26 min: 96–85% A, 26–28 min: 85–10% A, 28–32 min: 10% A, 32–34 min: 10–98% A, 34–39 min: 98% A) on a Thermo Scientific UltiMate 3000 Quaternary Analytical system with an FLD-3400RS fluorescence detector (excitation λ = 428 and emission λ = 525). The column was kept at 30°C to improve performance and reproducibility. Quantification was done by comparison to known weight of standard disaccharides (Iduron, UK), mock-treated in the same buffers and enzymes as the samples in each series of runs.

### Histological analysis

Dissected embryos at stages E16.5 and E19.5 were immersed in buffered 4% formalin for 3 or 7 days, respectively. Lungs from E18.5 embryos were submerged in fixative overnight. After fixation the embryos were dehydrated, paraffin embedded and subsequently serial sections of 5μm were obtained.

### Immunofluorescence staining

Paraffin sections with 5μm thickness from lungs of adult mice and E18.5 embryos were rehydrated and subsequently allowed to equilibrate for 15min in PBS and, after antigen retrieval treatment, the sections were blocked in 2% goat serum and 0.05% Tween/PBS for 1h. The sections were incubated with rabbit anti-Pro surfactant Protein C (pro-SPC, Abcam, ab90716, dilution 1:1000) overnight at 4^°^C. Next, the tissues were rinsed in PBS and then incubated for 2 h with Alexa Fluor-488 goat anti-rabbit secondary antibody (A-11008, Invitrogen, dilution 1:200). Lastly, the slides were incubated with DAPI for 15 min and mounted with VECTASHIELD Mounting Media (Vector Laboratories, H-1000) and visualized with Nikon Eclipse 80i microscope (Nikon Instruments, Japan).

### CXCL13 binding assay to mouse embryonic fibroblasts

Immortalized *Dse* ^+/+^ *Dsel* ^***-/-***^ and *Dse* ^-/-^ *Dsel* ^***-/-***^ mouse embryonic fibroblasts (MEFs) from E14.5 were generated according to established protocols and immortalized by transformation with recombinant simian virus 40 large T antigen carrying a *neo* gene [15].

To determine DS-epimerase-dependent binding, 2x10^5^ MEFs were incubated, while rotating, with 100 μl of CXCL13 (10 μg/mL) (R&D Systems) at 4°C for 90 min in RPMI 1640 medium containing 20 nM N-2-hydroxyethylpiperazine-N′-2-ethanesulfonic acid and 1% bovine serum albumin. Ligand concentration used represents the minimal concentrations required to saturate binding to control MEFs. Subsequently, cells were washed and incubated with rat anti-mouse CXCL13 (R&D Systems) to determine binding to the cells as measured by flow cytometry.

### Analysis and isolation of secondary lymphoid tissues of E19.5 embryos

Pregnant females were euthanized at E19.5, and embryos were collected and examined macroscopically by 2D-immunofluorescence or by flow cytometry. To assess lymphoid organ development, the embryos were autopsied at E19.5 and macroscopic images were taken with an Olympus (SZ61) dissection microscope (non-graded 0.67–4.5 × optical zoom) fitted with a digital camera. The images were processed using Adobe Illustrator and Photoshop CS4 (Adobe systems, San Jose, USA).

For immunofluorescence, whole embryos were frozen in TissueTek (Sakura Finetek Europe B.V., Alphen aan den Rijn, the Netherlands), and serial 7 μm cryosections were cut and fixed in dehydrated acetone for 5–10 min and air-dried for an additional 10 min. Fluorescent staining was performed essentially as described [10]. In brief, sections were pre-incubated with PBS containing 5% (v/v) normal mouse serum for 10 min, followed by a 45 min incubation with primary antibody and a subsequent 30 min incubation with an Alexa Fluor- (Invitrogen Life Technologies) or Cy3- (Sigma-Aldrich) labeled conjugate, where necessary. Fluorescent tissue was analyzed using a Zeiss fluorescent microscope (AXIO Imager.D2, Carl Zeiss, Germany), captured with ZEN 2 pro software (version 2.0.0.0, Carl Zeiss) and processed with Adobe CS4.

For flow cytometry analysis, mesenteric lymph nodes (MLNs) were dissected from E19.5 embryos and enzymatic digestion was performed as described [16]. After single cell suspensions were obtained, each individual MLN was incubated with primary antibodies for CD45, CD3 and CD4 to identify lymphoid tissue inducer (LTi) cells.

### Preparation of whole mount E13.5 mouse embryos for light sheet microscopy

Embryos were isolated and fixed in 0.4% paraformaldehyde (Aurion, Electron Microscopy Sciences) overnight at 4°C and prepared for light sheet microscopy essentially as described in detail in [17]. In short, after fixation, embryos were washed three times in PBS for 30 min and subsequently dehydrated and rehydrated in successive 30 min washes of 75%, 100%, 75%, 50% and 25% methanol. To minimize non-specific staining and to induce further permeabilization, tissues were incubated in 1% skim milk (Bio-Rad Laboratories B.V., Veenendaal, the Netherlands) and 0.4% TritonX-100 (Sigma-Aldrich) in PBS (PBS-MT) (v/v) twice for 1 hour at room temperature (RT). Embryos were then incubated for 3 days with freshly prepared PBS-MT containing primary conjugated antibodies, whilst rotating at 4°C in the dark. After incubation, embryos were washed three times for 1h and rinsed in PBS with 0.4% Triton X-100 (v/v). For easy handling, embryos were embedded in 2% low-melting agarose (Cambrex Bio Science Rockland, Rockland, USA). For clearing, embedded embryos were dehydrated in methanol series of 25%, 50%, 75%, 95%, 100% and 100%, each step for 1h. The clearing solution consists of 1:2 (v/v) benzyl alcohol: benzyl benzoate (BABB, both from Sigma-Aldrich). First, embryos were incubated in 50% BABB in methanol overnight at RT, rotating. After careful removal of all solutions, the embedded embryos were cleared in BABB at RT for at least 8h, rotating. This step was repeated with freshly made BABB when tissues were not sufficiently transparent. Embryos were stored for longer periods in freshly prepared BABB at RT in the dark.

After immunofluorescence whole mount staining, optically cleared E13.5 mouse embryos were imaged on a LaVision Ultramicroscope (LaVision BioTec, Bielefeld, Germany). Stacks were captured with a step size of 4 μm at the indicated magnifications. The 3D reconstruction and the analysis of ultramicroscope images were performed by applying IMARIS software (Version 8.0.1, Bitplane).

## Results

### Generation of dermatan sulfate epimerase-1 and -2 double knockout mice

Two mouse strains carrying the functionally inactivated *Dse* or *Dsel* gene, resulting in complete loss of dermatan sulfate epimerase-1 (DS-epi1) or -2 (DS-epi2) activity, respectively, have previously been generated in our laboratory [5, 7]. DS-epi1 knockout (KO) mice are viable in a mixed C57BL/6-129/SvJ genetic background but are perinatally lethal in a pure C57BL/6 or NFR genetic background [6]. DS-epi2^-/-^ mice were previously analyzed in a mixed C57BL/6-129/SvJ genetic background. The generation of *Dse* ^-/-^/*Dsel* ^-/-^ double KO (DKO) mice was performed as well in a mixed genetic background, and *Dse* ^+/-^ *Dsel* ^*-/-*^ parents derived from single KO mating were used for subsequent breeding. The choice of *Dsel* ^-/-^ parents was dictated by the observation that DS-epi2 overall contributes significantly less to the production of IdoA, and because DS-epi2 inactivation does not result in apparent phenotypic changes [7].

### Dermatan sulfate is completely absent in DKO mice

**Fig 1:**
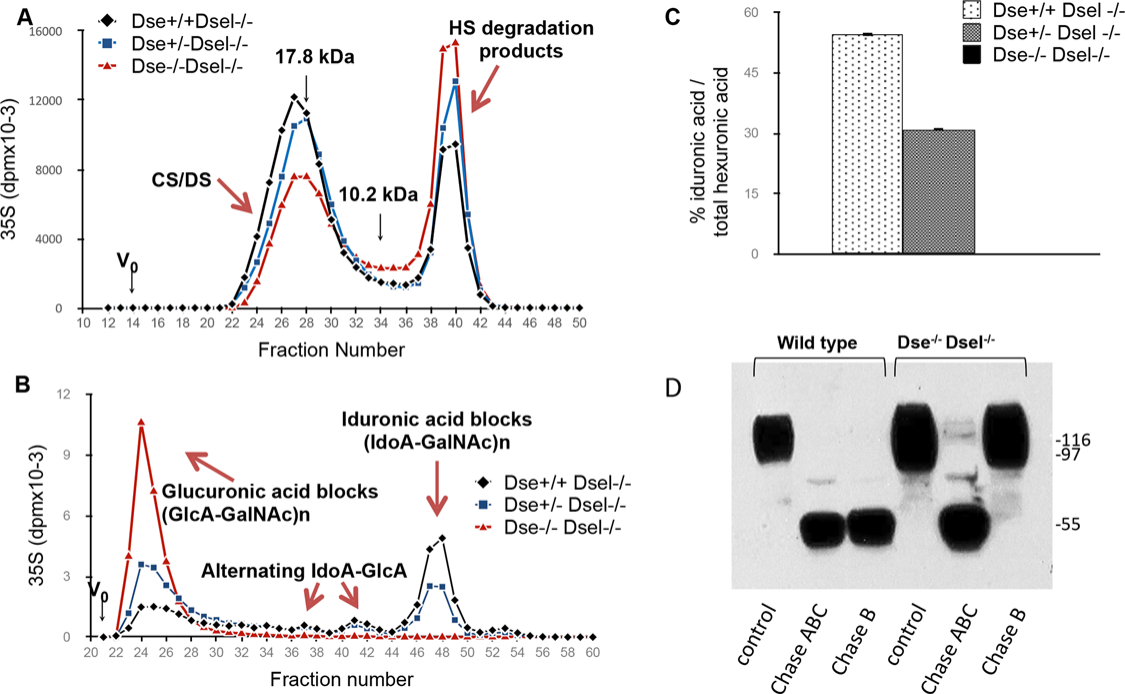
DKO mice are DS-free. Two-day-old pups were metabolically labeled with ^35^S sulfate. Skin decorin CS/DS was extracted and purified (A-C). A) CS/DS chains were analyzed on a size-permeation Superose 6 column. The HS degraded products, which were proportionally increased in the DKO mice, derive from the HS-PGs which co-eluted with decorin in the size permeation column used for PGs separation (see “Material and methods”). B) Split products obtained after chondroitinase B treatment were size separated on a Superdex Peptide column and C) the iduronic acid content was calculated. The iduronic acid analysis was conducted in duplicate samples. D) Skin PGs were extracted from E19.5 embryos of different genotypes. Decorin was stained before and after chondroitinase ABC and B treatment.

To verify the level of IdoA in DKO mice, two-day-old pups were injected with ^35^S-sulfate. Labeled decorin and biglycan were isolated from the skin, as these are the CS/DS-PGs with the highest level of IdoA in their chains [18], and were used to determine length and IdoA content of the polysaccharide chains. No difference in chain length was observed in the DKO mice compared to *Dse+/+/Dsel-/-* and *Dse+/-/Dsel-/-* mice, i.e. CS/DS from different genotypes were equally distributed in the same fractions (Fig 1A). Size separation of the split products obtained after chondroitinase B treatment, which only cleaves CS/DS chains at the iduronic acid~N-acetyl-galactosamine linkages, revealed complete absence of IdoA-dependent cleavage of chains obtained from DKO mice, implying total loss of epimerase activity (Fig 1B). We observed a gene dosage effect of *Dse* as monoallelic DS-epi1 (*Dse* ^*+/-*^ */Dsel* ^*-/-*^) expression resulted in only 31% of IdoA of total hexuronic acid residues, compared to 54% in mice with two functional *Dse* alleles (*Dse* ^*+/+*^ */Dsel* ^*-/-*^; Fig 1C). CS/DS extracted from the whole pups (minus skin) was similarly analyzed (data not shown). Again, chain length was unaffected. IdoA was 8.4% of total hexuronic acid in chains from the *Dse* ^*+/+*^ */Dsel* ^*-/-*^ mice and was reduced to 5.6% in *Dse* ^+/-^ *Dsel* ^***-/-***^ chains, and CS chains from DKO pups were also not cleaved by chondroitinase B. Another methodological approach to show the absence of IdoA in DKO mice was to extract PGs from whole skin of E19.5 embryos and to visualize decorin by Western blotting after treatment with chondroitinase ABC, which completely degrades the CS/DS chain to disaccharides irrespective of the epimer configuration, compared to digestion with chondroitinase B only. Decorin is degraded to its core protein by chondroitinase B when extracted from *Dse* ^+/+^/*Dsel* ^+/+^, *Dse* ^+/+^/*Dsel* ^-/-^ and *Dse* ^+/-^/*Dsel* ^-/-^ skin (the latter data not shown for brevity), but is unaffected when extracted from DKO skin (Fig 1D).

Together, our results show that monoallelic expression of DS-epi1 results in haploinsufficiency, revealed by a drop of IdoA content in the absence of DS-epi2. In addition, we demonstrate with different approaches that no IdoA could be detected in DKO mice, which can now be considered as a DS-free *in vivo* model.

### The composition of the chondroitin sulfate chain is altered in DKO mice

**Fig 2:**
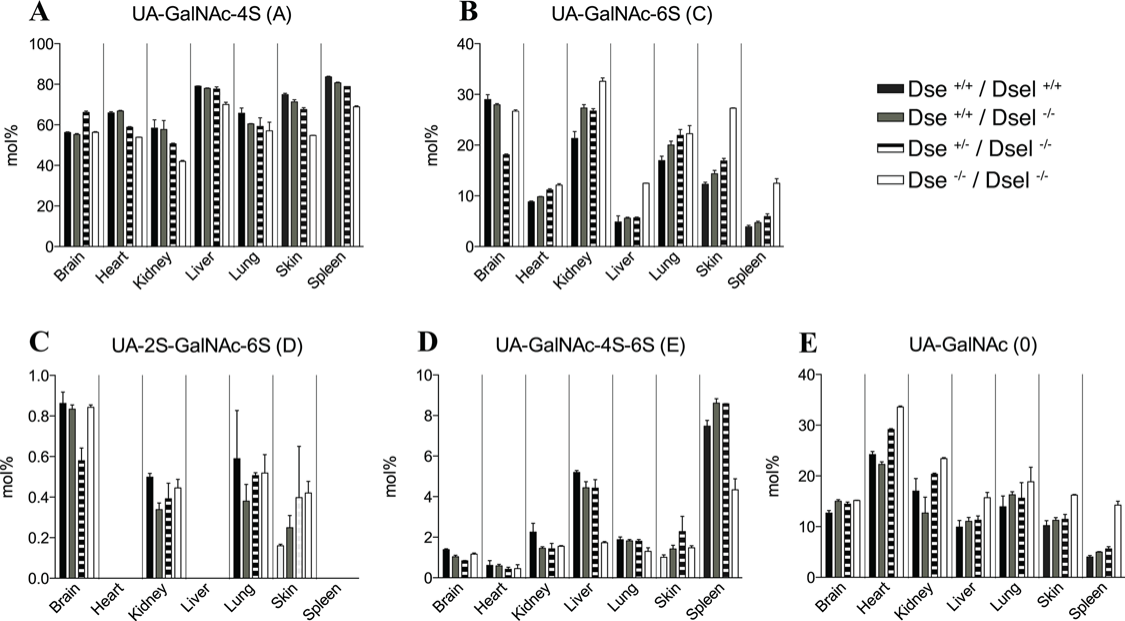
Disaccharide fingerprint of CS/DS from different organs of mice with decreasing epimerase expression. A-E) Disaccharides from different E19.5 organs were obtained after chondroitinase ABC cleavage, followed by AMAC-labeling and HPLC-separation and quantification. A, C, O, D and E units (mol%) are shown in the bar diagrams for the brain, heart, kidney, liver, lung, skin and spleen. Data are averages +/- s.e.m., n = 2.

To analyze the effect on the overall structural composition of CS/DS chains as a result of gradual decrease in gene dosage of the two DS epimerases, four genotypes were analyzed, *Dse* ^+/+^/*Dsel* ^+/+^, *Dse* ^+/+^/*Dsel* ^-/-^, *Dse* ^+/-^/*Dsel* ^-/-^ and *Dse* ^-/-^/*Dsel* ^-/-^, representing four, two, one, and null epimerase alleles, respectively. GAGs from E19.5 brain, heart, kidney, liver, lung, skin, and spleen were prepared from each genotype and fluorescent-labeled disaccharides were obtained after chondroitinase ABC treatment and quantified (Fig 2A–2E and S1 Table). The amount of CS/DS extracted from the different organs was comparable among the genotypes. The yield from DKO organs, expressed as ng CS-DS/mg dry tissue, generally varied less than 30% compared to the *Dse* ^+/+^/*Dsel* ^+/+^ organs, without genotype-dependent trend (data not shown). The overall comparison revealed differences in chain composition depending on the tissue, the gene dosage and the disaccharide unit. By and large in *Dse* ^+/+^/*Dsel* ^-/-^ mice, no major changes in disaccharide distribution were noted compared to *Dse* ^+/+^/*Dsel* ^+/+^ mice. The effect of a decrease in DS-epi1 expression was observed by comparison of the *Dse* ^+/+^/*Dsel* ^+/+^, *Dse* ^+/-^/*Dsel* ^-/-^ and *Dse* ^-/-^/*Dsel* ^-/-^ genotypes. In all tissues but the brain, similar changes were observed for the major 4- and 6-monosulfated structures (A unit, Fig 2A; C unit, Fig 2B, respectively) and the non-sulfated structure (O unit, Fig 2E). The A unit decreased in parallel with decreasing DS-epi1 expression, while the O and C units increased. No major changes were observed in the brain, which can be attributed to the very low content of IdoA in this tissue [7, 19]. The less predominant disulfated disaccharides were also altered upon changes in the expression of the DS epimerases. The D unit (Fig 2C) was relatively unaffected in all tissues but skin, where it increased upon downregulation of the DS epimerases. The 4- and 6-sulfated N-acetyl galactosamine-containing structure (E unit, Fig 2D) was unaffected in the tissues where the abundance was low, but was decreased in the DKO tissues, liver and spleen, with a high abundance of this unit. Finally, the 2-4-disulfated disaccharides (B unit, S1 Table) disappeared completely in the DKO tissues.

Altogether, the data indicate that CS/DS has a unique composition in each organ and is profoundly changed in DKO mice. This underscores the importance of strict regulation of tissue-specific expression of the DS epimerases to generate the required structures in CS/DS and that loss of activity could result in altered biological properties affecting a wide range of cellular processes.

### Macroscopic developmental analysis of DKO mice

During the first months of the colony few DKO mice (n = 3) survived after the perinatal period. Two mice, characterized by reduced weight (18% and 51% reduction compared to the average of their *Dse* ^+/+^/*Dsel* ^-/-^ and *Dse* ^+/-^/*Dsel* ^-/-^ littermates) died at seven weeks of age and were lost to analysis. One DKO mouse was analyzed at day 16. This mouse presented a tail with several kinks and a 38% body weight reduction compared to its littermates (S1 Fig). All of the major organs were necroscopically and histologically normal. Interestingly, subsequent breeding rounds resulted in no DKO mice of the 200 genotyped mice at weaning. This observation can be explained by genetic drift; i.e., the accumulation of an unfavorable allelic mixture in our small colony. Instead, perinatal mortality was observed; i.e., corpses or body remains of eaten pups within 48 hours after birth. For this reason, embryos from mid- (E13.5) to late- (E16.5-E19.5) gestational stages were studied. The size and weight of DKO embryos from all gestational stages did not differ from the littermate controls (data not shown). As shown in Table 1, from 174 embryos genotyped, regardless of the developmental stage, the DKO embryos occurred at Mendelian ratios.

**Table 1:** DKO (*Dse* ^-/-^/*Dsel* ^-/-^) embryos were found at Mendelian ratios.

Embryonic	N	*Dse* ^*+/+*^ *Dsel* ^*-/-*^	*Dse* ^*+/-*^ *Dsel* ^*-/-*^	*Dse* ^*-/-*^ *Dsel* ^*-/-*^
day		(%)	(%)	(%)
**E19.5**	49	16 (33%)	21 (43%)	12 (24%)
**E18.5**	80	29 (36%)	35 (45%)	16 (19%)
**E16.5**	24	5 (18%)	11 (50%)	8 (32%)
**E13.5**	21	3 (19%)	11 (50%)	7 (31%)

**Fig 3:**
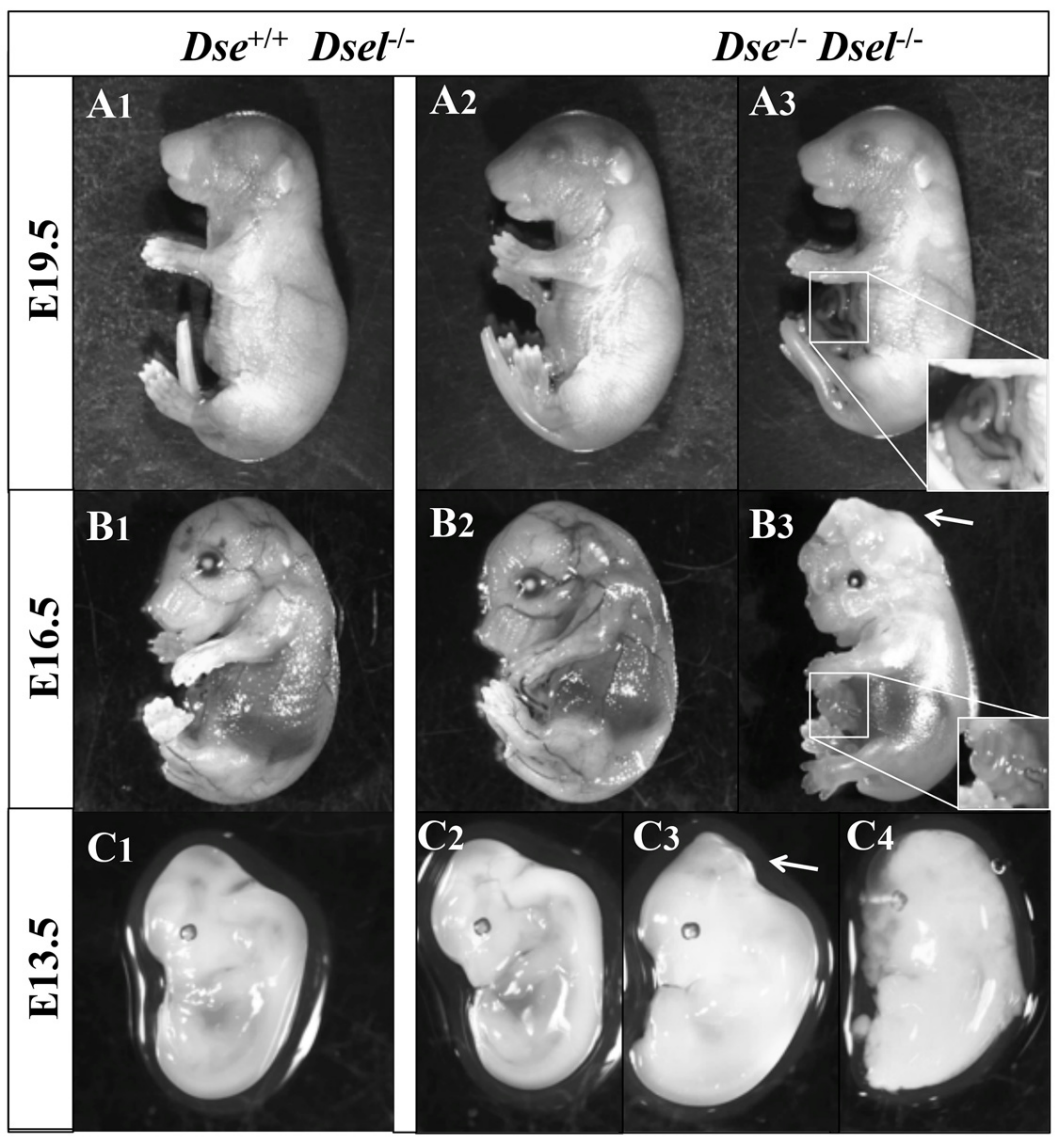
Developmental defects of DKO embryos. A1, B1, C1) *Dse* ^+/+^/*Dsel* ^-/-^ embryos. A2, B2, C2) DKO embryos with no obvious phenotype. A3, B3, C3-4) DKO embryos with umbilical hernia (A3, B3, magnified in squares) and exencephaly (B3, C3, arrows) C4 is a poorly developed embryo. The kinked tail phenotype can be seen in A3.

Overall, from macroscopic observation of 116 E13.5-E19.5 embryos, the predominant findings were abdominal wall closure defects in 43% of the DKO embryos, kinked tail in 29%, exencephaly in 7% (Table 2 and Fig 3). Therefore, the penetrance of each phenotype varied greatly, resulting in embryos ranging from macroscopically normal to severely affected.

**Table 2:** Abdominal wall defects, kinked tails, and exencephaly were present at variable penetrance in DKO embryos.

Embryonic day	Genotype	N	Abdominal wall defects	Kinked Tail	Exencephaly
**E19.5**	***Dse*** ^***+/+***^ ***Dsel*** ^***-/-***^	5	0	0	0
	***Dse*** ^***+/-***^ ***Dsel*** ^***-/-***^	11	0	0	0
	***Dse*** ^***-/-***^ ***Dsel*** ^***-/-***^	5	1	2	0
**E18.5**	***Dse*** ^***+/+***^ ***Dsel*** ^***-/-***^	19	0	0	0
	***Dse*** ^***+/-***^ ***Dsel*** ^***-/-***^	21	2	1	0

	***Dse*** ^***-/-***^ ***Dsel*** ^***-/-***^	9	5	4	0
**E16.5**	***Dse*** ^***+/+***^ ***Dsel*** ^***-/-***^	5	1	0	0
	***Dse*** ^***+/-***^ ***Dsel*** ^***-/-***^	11	4	1	0
	***Dse*** ^***-/-***^ ***Dsel*** ^***-/-***^	9	6	2	1
**E13.5**	***Dse*** ^***+/+***^ ***Dsel*** ^***-/-***^	3	0	0	0
	***Dse*** ^***+/-***^ ***Dsel*** ^***-/-***^	11	0	0	0
	***Dse*** ^***-/-***^ ***Dsel*** ^***-/-***^	7*	0	0	1


**Fig 4:**
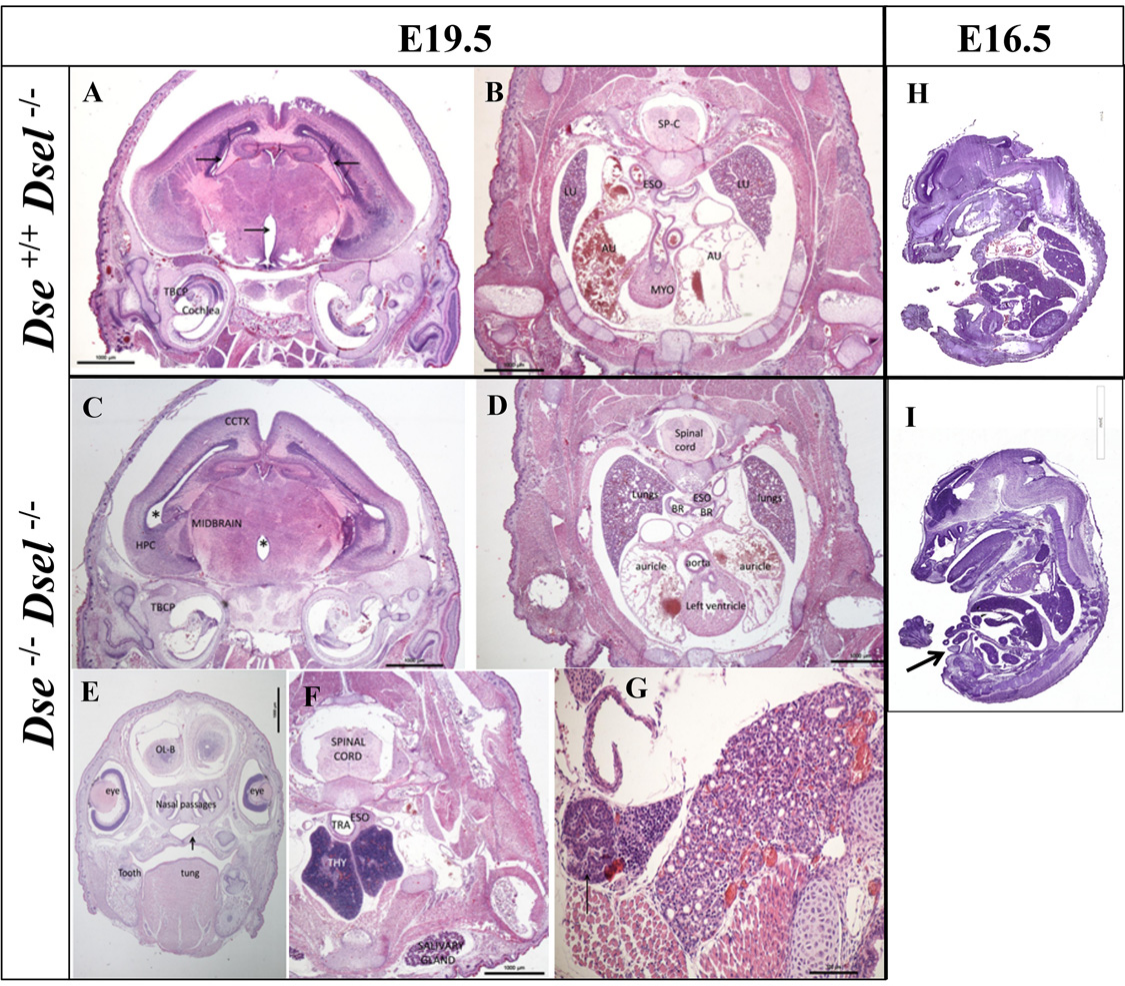
Histological staining of DKO embryos. Sections from three E19.5 embryos showing no macroscopic abnormalities and from two E16.5 embryos with umbilical hernia were HE stained. Transversal sections of E19.5 brain (A, C), lung/heart/spinal cord (B, D), tongue/palate (E), and thymus (F, G). Whole E16.5 embryo sagittal sections (H, I). An umbilical hernia can be seen in the DKO embryo (I, arrow). Bars = A-F = 1000 μm, G = 100 μm, H = 2 mm, I = 3 mm.

* One embryo had developed poorly (see Fig 4C4).

### DKO embryos reveal no aberrations in major organs

To find possible organ defects, detailed histological evaluation of E19.5 *Dse* ^+/+^/*Dsel* ^-/-^ (Fig 4A and 4B) and DKO embryos (Fig 4C–4G) which appeared macroscopically normal was performed. In all organs, no obvious aplasia, hemorrhage, necrosis, inflammation or other type of lesion were observed. The brain (thalamus, hypothalamus, cerebral hemispheres and ventricles, cerebellum, eyes, and pituitary gland), spinal cord, liver, and lungs appeared normal when compared to controls (Fig 4A and 4C and S2A and S2B Fig). The lungs showed atelectasis, which can be expected at this embryonic stage. In addition, paraffin sections of DKO embryo lungs were stained with pro-surfactant protein C (pro-SP-C), which is a marker for Alveolar type II cells. Comparisons of DKO and *Dse* ^+/+^/*Dsel* ^-/-^ lungs did not reveal any differences in immunofluorescent staining (S2C and S2D Fig). Also, when whole E18.5 embryos were stained with Alcian Blue/Alizarin Red staining, no differences in bone/cartilage proportion were observed (data not shown). The airways (larynx, trachea, and bronchi) (Fig 4B–4D), the palate and the thymus (Fig 4E–4G) did not reveal any distinct discrepancies. As depicted in Fig 4H and 4I, DKO E16.5 embryos did not show additional histological abnormalities other than the umbilical hernia (Fig 4H–4I).

In conclusion, while we demonstrated profound changes in the composition of the CS chains in major organ compartments, including the kidneys, liver and lungs, upon complete loss of DS, detailed analysis did not show any evidence of detrimental changes that could explain the perinatal lethal phenotype in DKO mice.

### Binding of CXCL13 to DKO MEFs is severely impaired

**Fig 5:**
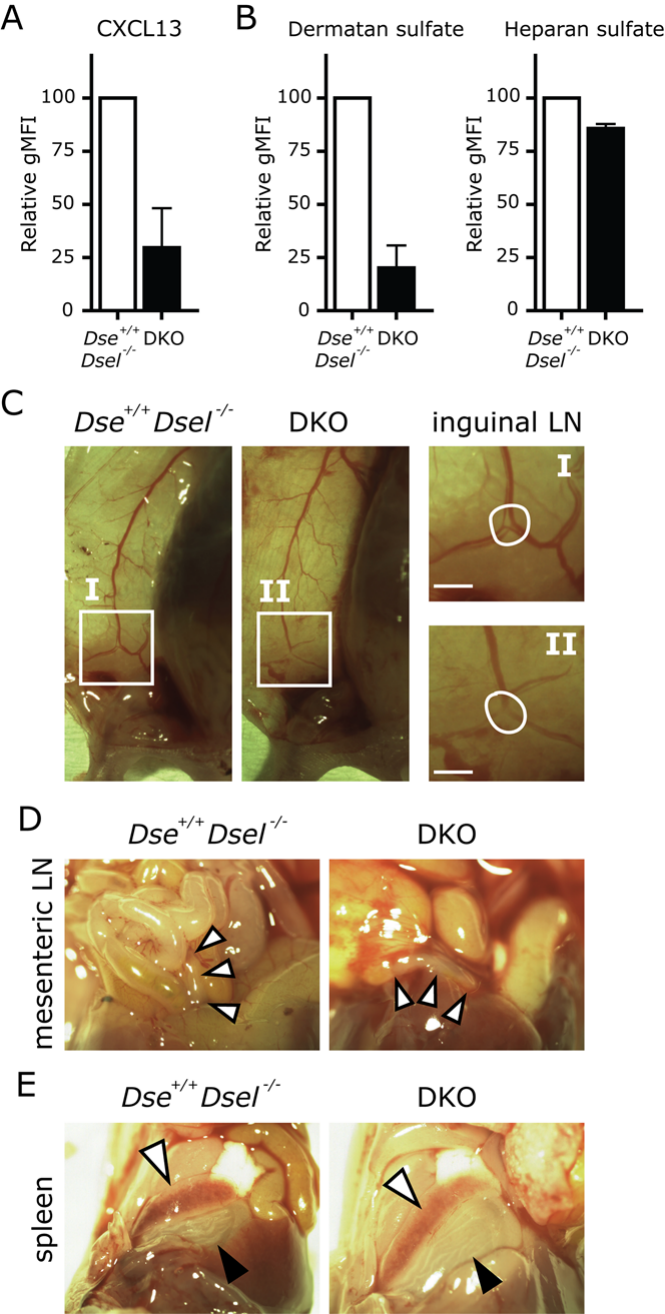
Impaired CXCL13 binding does not prevent lymph node formation in DKO embryos. A) Mouse embryonic fibroblasts (MEFs) were incubated with CXCL13 and binding was measured by flow cytometry. Relative binding to cell surfaces is shown as geometric mean fluorescent intensity with background stain subtraction of secondary antibody only (gMFI). B) Relative expression of cell surface dermatan sulfate and heparan sulfate is shown as measured by GD3A12 [11] and HS4E4 [12] antibody binding, respectively, with background stain subtraction of secondary antibody only. The bars are averages +/- s.e.m., n = 3 (A, B). C) Blood vessel branching in control and DKO skin is depicted. In I and II, inguinal lymph node (LN) primordia are shown (encircled) as enlargement (squares) at the site of typical bifurcation of the blood vessel. D, E) Mesenteric LNs and spleen in *Dse* ^*+/+*^ */Dsel* ^*-/-*^ and DKO embryos are highlighted by white arrow heads. The black arrow heads point to the stomach (E), which typically lies adjacent to the spleen. The bars are 100 μm (C).

Because it was shown that mice lacking heparan sulfate (HS) epimerase activity displayed a variety of defects in lymphoid organ development and lymphocyte function [10, 20], and high DS epimerase activity was observed in the spleen [5], the binding of CXCL13 to MEFs generated from *Dse* ^*+/+*^ */Dsel* ^*-/-*^ and DKO embryos was analyzed. This is highly relevant, as CXCL13 is an important chemokine in the initiation of secondary lymphoid organ development and lymphocyte migration and homing [21]. Interestingly, the binding of CXCL13 to DKO MEFs was severely reduced (Fig 5A), which was paralleled by the loss of cell surface DS, while cell surface HS was unaffected (Fig 5B and 5C). These results suggest that cell surface binding of CXCL13 requires DS, and that the impaired binding to DS could result in aberrations in lymphoid organ development.

### Secondary lymphoid organ development and cellular organization is unaffected in DKO mice

Prompted by the loss of CXCL13 binding to DKO MEFs, the development of secondary lymphoid organs in *Dse* ^*+/+*^ */Dsel* ^*-/-*^ and DKO embryos (E18.5-E19.5) was specifically analyzed. These studies included the peripheral (pLNs) and mesenteric lymph nodes (mLNs) and the spleen. Initially, *Dse* ^*+/+*^ */Dsel* ^*-/-*^ (n = 5) and DKO (n = 5) E19.5 embryos were harvested and macroscopically examined. In all DKO embryos, inguinal (iLN), brachial (bLN), and axillary (aLN) LNs could be readily detected (Fig 5C and data not shown). Further, apparent normal blood vessel branching and outgrowth was observed, as exemplified by the bifurcation of the blood vessel at the location where iLNs normally develop (Fig 5C). In addition, mLNs and spleens were easily identified and showed no aberrations in localization or size (Fig 5D and 5E).

**Fig 6:**
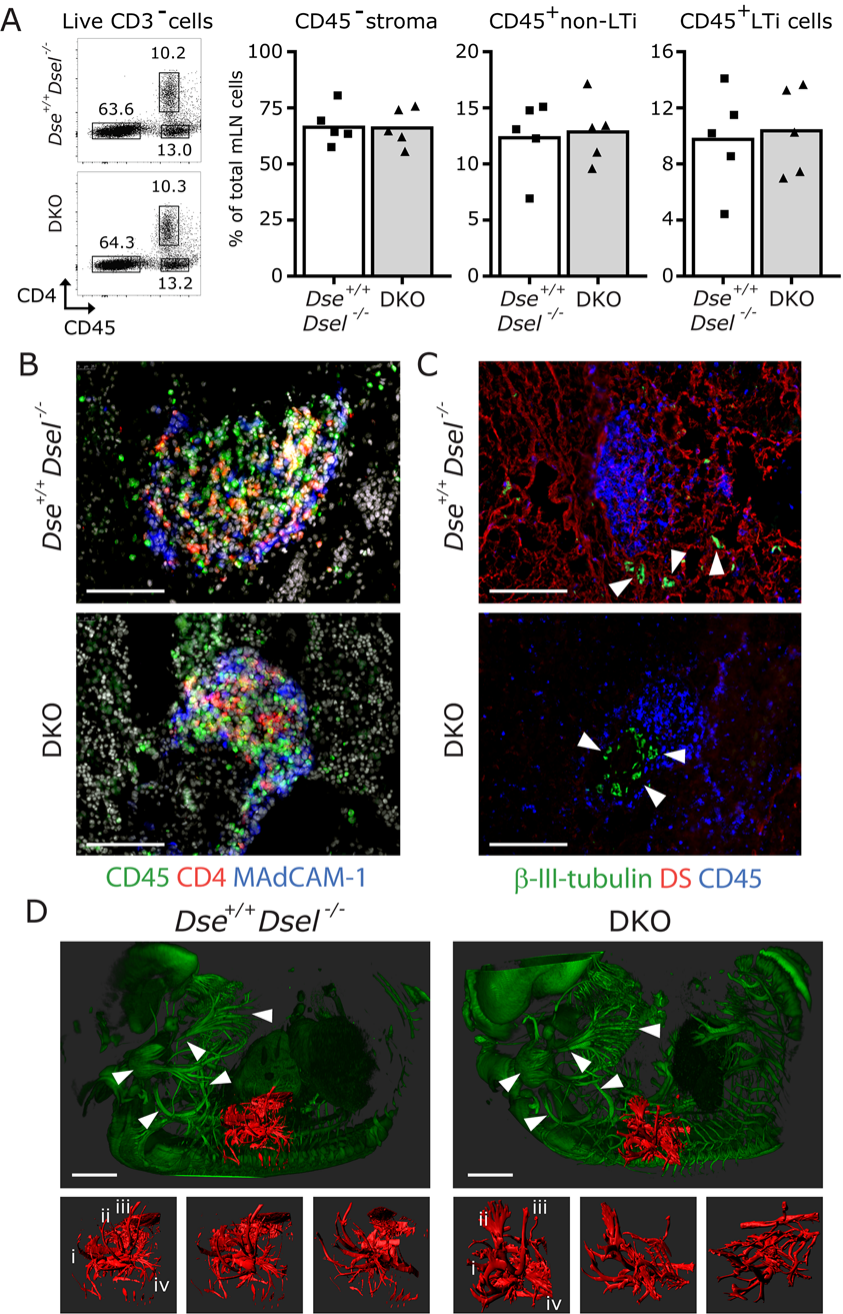
Normal percentage of lymphoid tissue inducer cells correlate with normal development of lymph node primordia and nerve fiber outgrowth in DKO embryos. A) Percentage of CD45- stroma, CD45+ non-LTi cells and LTi cells is shown. FACS dot plots are representative of the averages shown in the bar graphs. B) CD4+CD45+ LTi cells were readily detected, surrounded by CD45-MAdCAM-1+ stromal cells. C) Serial sections of B revealed that class-III β-tubulin positive nerve fibers lie in close proximity to the CD45+ cell clusters. Both surround the lymph node and are found within the primordia. Dermatan sulfate (DS, red) positive fibers are found in *Dse* ^*+/+*^ */Dsel* ^*-/-*^ embryos, which are absent in DKO mice. D) No gross aberrations were found (n = 3) in nerve fiber outgrowth as shown by representative 3D light sheet microscopy for class-III β-tubulin (green). Extrusions at the location of the right arm are highlighted in red and depicted in three different orientations, and show that a similar type of branching is present in *Dse* ^*+/+/*^ *Dsel* ^*-/-*^ and DKO E13.5 embryos, indicated with I-IV. White arrow heads point to comparable large nerve fibers that are typically found at these locations. The bars are 100 μm (B, C) and 400 μm (D).

Despite the loss of CXCL13 binding to DKO MEFs, the development of LNs was unaffected. For this reason, the mLNs were isolated to determine the amount of lymphoid tissue inducer (LTi) cells, which are crucial for the formation and organization of LNs [21]. At this stage of LN development, LTi cells can be identified by the expression of CD4 and CD45, while lacking the expression of CD3. After isolation and enzymatic digestion, LTi cells of mLNs were analyzed by flow cytometry, which revealed that the percentage of LTi cells of the total LN population (approximately 10%) was similar in DKO mice compared to *Dse* ^*+/+*^ */Dsel* ^*-/-*^ embryos (Fig 6A), and in line with previous observations [22]. In addition, the percentages of CD45- stromal cells and CD45+ hematopoietic cells, other than LTi cells, were unaltered.

In accordance with these results, multiple LN primordia in E18.5 DKO embryos (n = 2) at specific locations were identified when whole body serial cryosections were analyzed. To assess pLN development in more detail, immunofluorescence for CD4, CD45 and MAdCAM-1 on these pLN primordia was applied. Normal LTi clusters (CD4+CD45+) surrounded by stromal cells (CD45-MAdCAM-1+) were apparent (Fig 6B), which is the typical organization of late stage (E18.5) pLNs. In addition, neuronal class-III β-tubulin-expressing nerve fibers adjacent to these LTi clusters were also identified (Fig 6C). This suggests that nerve outgrowth and function is normal in DKO embryos, allowing the initial expression of CXCL13 by LN stromal cells to attract LTi cells, triggered by nerve fibers through the expression of retinoic acid [23]. In support of this conclusion, total body 3D light sheet microscopy on E13.5 DKO embryos (n = 3) did not reveal any gross abnormalities in nerve fiber outgrowth in general, or at typical locations of LN initiation (Fig 6D and data not shown).

Together, the data show that all DS-free embryos generate pLNs, mLN, and spleens at the correct locations, and develop a nerve fiber network of normal appearance, which does not affect LN formation and allows for clustering of expected percentages of LTi cells and LN stromal cells resulting in undisturbed LN development up to gestational day E19.5.

## Discussion

Three enzymes are specific for DS biosynthesis: the two epimerases DS-epi1 (encoded by the *Dse* gene) and DS-epi2 (encoded by *Dsel*), which convert GlcA into its epimer IdoA, and the 4-O-sulfotransferase D4ST1 (encoded by *Chst14*), which adds one sulfate group to the GalNAc residue adjacent to IdoA. This step locks the epimer conversion and allows the formation of long (IdoA-GalNAc-4-S)_n_ blocks [24]. DS-epi1 is the main epimerase *in vivo* and in several cell types, including fibroblasts and smooth muscle cells [7, 25]. Interestingly, the brain, which contains very low level of iduronic acid, is the only tissue where DS-epi2 predominates [7, 19]. Based on the insight gained from the *Dse* ^*-/-*^ and *Dsel* ^*-/-*^ single KO mouse models [5–7] regarding the importance of DS in development and tissue integrity, and the suggested redundancy of enzymatic function between the two DS epimerases, we were prompted to develop and study a mouse model with both epimerases inactivated. Our aim was bidirectional: one part was focused on the detailed structural characterization of CS/DS chains in different organs and the induced changes in DKO tissues; the other part focused on the description of prenatal development and organogenesis in the absence of DS.

The data demonstrate that DS-epi1 and DS-epi2 are the sole epimerases. Hence, our DKO model is the first mouse that is completely DS-free. The analyses were conducted on skin decorin, known to have the highest content of IdoA [18] and on the complementary total body CS/DS, which both revealed a complete absence of IdoA. The relative contribution of the two epimerases to the biosynthesis of IdoA in skin decorin and the gene dosage effect is apparent comparing the different genotypes. The decorin CS/DS chain contained 67% of IdoA in *Dse* ^*+/+*^ */Dsel* ^*+/+*^, 67% in *Dse* ^*+/-*^ */Dsel* ^*+/+*^, 18% in *Dse* ^*-/-*^ */Dsel* ^*+/+*^ [5] 54% in *Dse* ^*+/+*^ */Dsel* ^*-/-*^, 31% in *Dse* ^*+/-*^ */Dsel* ^*-/-*^, and zero in the DKO newborn pups. Obviously, *Dse* is predominant and loss of one allele can be compensated only when *Dsel* is present. Whether D4ST1 deficiency would result in the complete absence of IdoA was not analyzed in the *Chst14* ^*-/-*^ mouse, and requires further investigation [26, 27].

Biochemical analysis revealed that the amount of CS/DS present in the WT and DKO organs was comparable, but a profound and tissue-dependent impact of the complete lack of IdoA was noted in the structural profile of the chains. The abundance of the major structural units was affected in DKO organs: the amount of mono 4-sulfated unit decreased while the abundance of mono 6-sulfated and the non-sulfated units increased. How these variations are accomplished by the CS/DS biosynthetic machinery is not known. We can speculate that competition between different enzymes and limiting substrate takes place during chain synthesis: lack of epimerases would prevent D4ST1 to act on GalNAc residues, which instead could be modified by the other three 4-O-sulfotransferases C4ST1-3, or by the 6-O-sulfotransferase C6ST1, or by escaping sulfation. The disulfated disaccharides, critical in several biological properties of CS/DS, are affected as well: the 4- and 6-disulfated E unit selectively decreases in DKO liver and spleen while the 2- and 6-disulfated D unit increases in spleen. The 2- and 4-disulfated B unit disappears in all tissues of the DKO mice, probably reflecting a greater affinity *in vivo* of the 2-O-sulfotransferase UST for IdoA compared to GlcA, as shown *in vitro* [28]. Not surprisingly, the changes in the structure result in functional abnormalities because IdoA-containing structures have been described to be essential, for instance, in binding to growth factors and morphogens [29].

Regarding the study of prenatal development and organogenesis in the absence of DS, we performed a phenotypic analysis of DKO mice in a mixed C57BL/6-129/SvJ genetic background. Most of DKO embryos showed abnormalities: 27% had a kinked tail, 40% an umbilical hernia and 7% exencephaly. In comparison, *Dse* ^*-/-*^ newborn mice in a pure NFR background, 100% had a kinked tail, 16% had umbilical hernia and 5% had neural tube defects (exencephaly, spinabifida and ringelschwanz) [6]. Deranged collagen structure could interfere with proper abdominal wall closure and could be the cause of the observed umbilical hernia. In humans, there are two common abdominal wall closure defects, omphalocele and gastroschisis, which have many similarities but also various differences [30]. Exencephaly, or anencephaly for humans, is a neural tube defect that causes impaired closure in the cranial portion of the neural tube early in development and leads to loss of brain tissue [31]. This study further supports the notion that this DS-free mouse model could be a useful genetic model for the human diseases. Nevertheless, further molecular studies are needed. The majority of embryological defects observed are not per se the cause of embryo death or resorption at the embryonic stages studied. In fact, we observed percentages of genotypes that correspond to Mendelian ratios at all gestational stages and the embryos that have been obtained after caesarian section at the latest gestational stage appeared to be alive and moving. Both abdominal closure and exencephaly can lead to neonatal death due to the exposure of tissues during parturition and could lead to cannibalization. However, a minority of the DKO embryos looked histologically normal, but nonetheless all newborn died during the first 48 hours after birth, except three mice that survived for several weeks and had greatly reduced body size. We examined the primary physiological processes that could lead to neonatal death, such as breathing and suckling [32]. The lungs of the DKO embryos were normally developed, and the palate looked normal as well. It may be assumed that mice die due to multisystem fragility and multiple organ failure. Macroscopic and histological analysis did not reveal any lesions or organ hypoplasia or agenesis. Lastly, no apparent congenital multiple contractures were observed.

Similar phenotypic variability has been recently described in the *Chst14* knockout model in the mixed C57BL/6-129/SvJ background in which all IdoA-containing blocks are abrogated. Most of these KO mice died between embryonic days E16.5 to E18.5, but 8% survived after birth. The viable *Chst14* ^−/−^ mice were smaller but otherwise normal and had a lifespan similar to their wild-type littermates. Lastly, similar to the *Dse* ^*-/-*^ mice, the skin of *Chst14* ^*−/−*^ mice was more fragile compared to *Chst14* ^+/+^ animals [26].

Recently, the first human pathological condition involving DS biosynthetic deficiency has been identified as a sub-type of Ehlers-Danlos syndrome coined ‘Musculocontractural Ehlers-Danlos syndrome’ (MC-EDS). The pathological features of MC-EDS are attributed to genetic mutations in the CHST14 (approximately 30 patients) or DSE (3 patients) genes. MC-EDS patients suffer from connective tissue fragility (joints, skin, internal organs), malformations (craniofacial features, congenital contractures) and congenital cardiovascular, gastrointestinal, renal, ocular and central nervous system defects [27, 33–35]. The reason for the more severe changes in patients compared to *Dse* ^-/-^ mice in the mixed C57BL/6-129/SvJ genetic background is not known. DSEL can also be the cause of human disease as it has been genetically linked to type II bipolar disorder [36]. No humans with double mutations in both DSE and DSEL have been described.

Because HS epimerization resulted in impaired lymphoid organ development and lymphocyte function [10, 20], and CXCL13 binding to DKO MEFs was severely affected, our attention was drawn to lymphoid organogenesis, which we anticipated to be significantly affected, especially because DS epimerase activity in the spleen is highly abundant [5] and the immunostaining of LN primordia showed abundant DS in control embryos. However, analysis of secondary lymphoid organ development in DKO embryos uniformly revealed normal localization, population and outgrowth of the splenic and lymph node primordia. This suggests that chemokines, including CXCL13, which are critical for the attraction of the first LTi cells, are adequately produced and maintained at proper locations independent of DS. This corroborates the findings in HS glucuronyl C5-epimerase (*Glce*) deficient mice, where widespread abnormalities were observed in the development of distinct primary and secondary lymphoid organs, while CS/DS synthesis was unaffected. Clearly, proper HS modification, as demonstrated by loss of *Glce* expression, is essential for correct lymphoid organogenesis, while DS is dispensable [10]. Recently, for LNs, it was demonstrated that initiation of LN development is controlled by adjacent neurons, which trigger the expression of CXCL13 in stromal cells through the production of retinoic acid [23]. Therefore, we performed whole body 3D light sheet microscopy for class-III β-tubulin on control and DKO E13.5 embryos to study nerve fiber development and outgrowth. This is especially interesting because CS is accepted as a major inhibitor of neuronal regeneration. However, in line with the findings in *Chst14* ^-/-^ mice, which lack IdoA-containing modules present only in DS [26], DKO mice revealed nerve fiber networks comparable to control littermates. In support of this conclusion, mesenteric LNs at E19.5 consisted of normal percentages of stromal cells and CD45+ cells, including similar percentages of LTi cells.

Overall, our study is the first report that specifically addresses the impact of the loss of DS on embryonic development. Our results showed that DS is crucial for neonatal survival, and revealed dispersed developmental aberrations like abdominal wall defects. Despite neonatal lethality, the data indicate that homeostatic organogenesis, for instance the development of secondary lymphoid organs, is unaffected in the absence of DS. Therefore, the described DS-free mice could be a useful model for human congenital disorders.

## Supporting Information

S1 Fig Sixteen-day-old DKO mouse has greatly reduced body size. (TIF)

S2 Fig Lungs from E18.5 DKO embryos appear normal (TIF)

S1 Table Disaccharide fingerprint of CS/DS from E19.5 organs from mice with decreasing epimerases expression. (XLSX)
