# Supplementary figures and images for: Dermatan Sulfate-Free Mice Display Embryological Defects and Are Neonatal Lethal Despite Normal Lymphoid and Non-Lymphoid Organogenesis
Source: PLoS One. 2015 Oct 21;10(10):e0140279. doi: 10.1371/journal.pone.0140279 (PMC4619018; doi:10.1371/journal.pone.0140279)

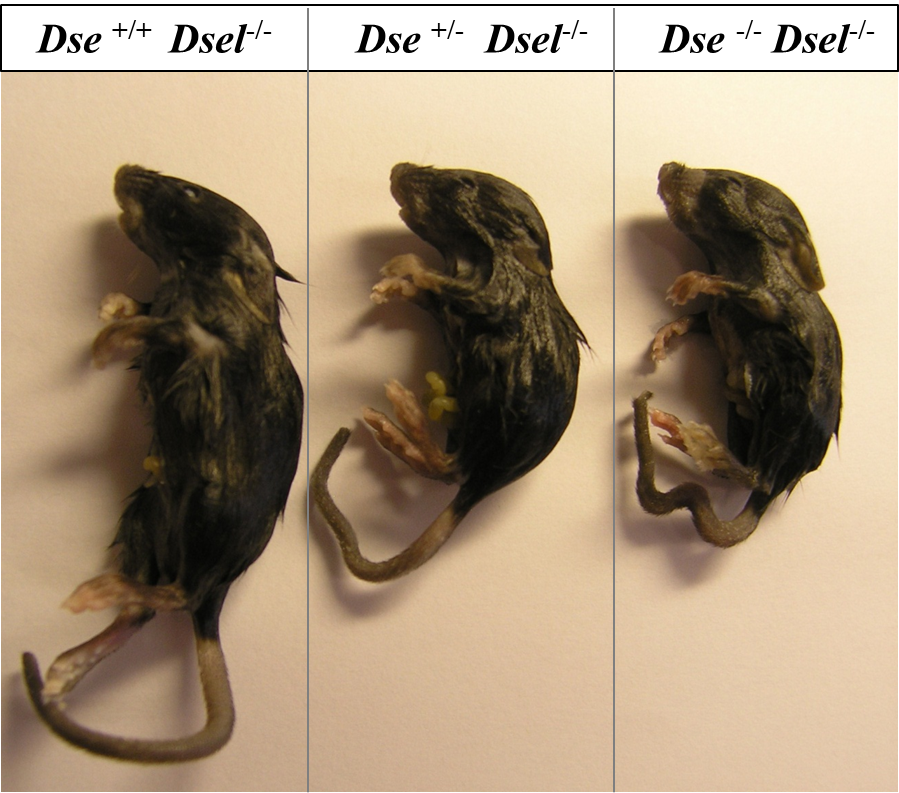

Supplement: S1 Fig — (TIF) [file pone.0140279.s001.tif]

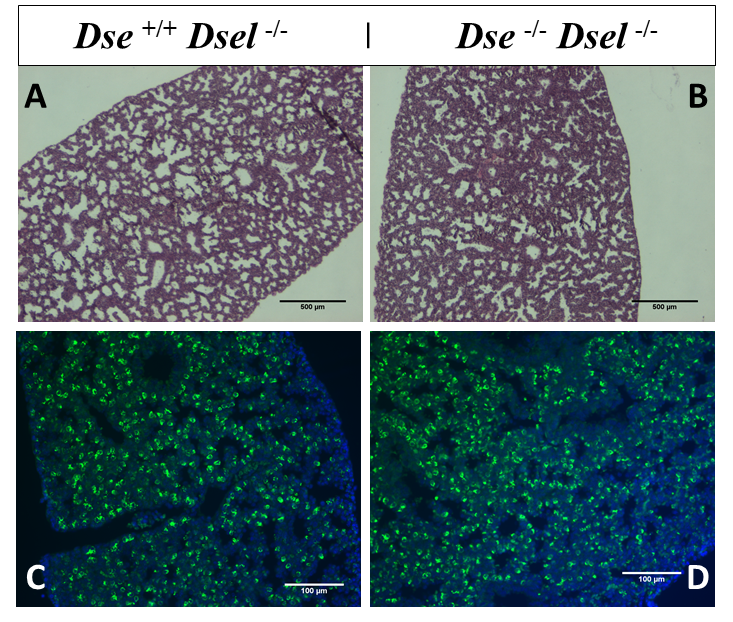

Supplement: S2 Fig — (TIF) [file pone.0140279.s002.tif]
